# Contribution of seagrass plants to CO_2_ capture in a tropical seagrass meadow under experimental disturbance

**DOI:** 10.1371/journal.pone.0181386

**Published:** 2017-07-13

**Authors:** Diana Deyanova, Martin Gullström, Liberatus D. Lyimo, Martin Dahl, Mariam I. Hamisi, Matern S. P. Mtolera, Mats Björk

**Affiliations:** 1 Seagrass Ecology & Physiology Research Group, Department of Ecology, Environment and Plant Sciences, Stockholm University, Stockholm, Sweden; 2 School of Biological Science, University of Dodoma, Dodoma, Tanzania; 3 College of Natural and Mathematical Sciences, University of Dodoma, Dodoma, Tanzania; 4 Institute of Marine Sciences, University of Dar es Salaam, Zanzibar, Tanzania; Università della Calabria, ITALY

## Abstract

Coastal vegetative habitats are known to be highly productive environments with a high ability to capture and store carbon. During disturbance this important function could be compromised as plant photosynthetic capacity, biomass, and/or growth are reduced. To evaluate effects of disturbance on CO_2_ capture in plants we performed a five-month manipulative experiment in a tropical seagrass (*Thalassia hemprichii*) meadow exposed to two intensity levels of shading and simulated grazing. We assessed CO_2_ capture potential (as net CO_2_ fixation) using areal productivity calculated from continuous measurements of diel photosynthetic rates, and estimates of plant morphology, biomass and productivity/respiration (P/R) ratios (from the literature). To better understand the plant capacity to coping with level of disturbance we also measured plant growth and resource allocation. We observed substantial reductions in seagrass areal productivity, biomass, and leaf area that together resulted in a negative daily carbon balance in the two shading treatments as well as in the high-intensity simulated grazing treatment. Additionally, based on the concentrations of soluble carbohydrates and starch in the rhizomes, we found that the main reserve sources for plant growth were reduced in all treatments except for the low-intensity simulated grazing treatment. If permanent, these combined adverse effects will reduce the plants’ resilience and capacity to recover after disturbance. This might in turn have long-lasting and devastating effects on important ecosystem functions, including the carbon sequestration capacity of the seagrass system.

## Introduction

Climax-stage ecosystems can generally cope with mild or occasional stress from factors such as light limitation and grazing pressure. In marine coastal environments, natural light-deprivation events are often temporary and caused by, for example, seasonal river runoff or heavy rain periods that reduce water transparency [1]. Most nearshore marine ecosystems are adapted to resist such short periods of light reduction, with no effects on species composition or ecosystem stability [2]. Mild grazing has been demonstrated to stimulate plant growth [3] and shallow-water coastal habitats such as seagrass meadows are adapted to low levels of grazing [4]. Intensive grazing, however, can have severe negative effects on many species, leading to shifts in species composition [5]. Prolonged periods of high-intensity stress in the marine environment could drive an ecosystem to a threshold at which change or adaptation will result, but with negative effects on important ecosystem services provided by the ecosystem [6,7]. There is, however, a clear lack of field studies assessing multi-intensity impacts of relevant stressors such as light limitation and grazing on seagrass meadow productivity linked to carbon sink capacity.

Seagrass habitats are considered one of the most productive marine ecosystems, well recognized for their provision of numerous highly valuable ecosystem services [8]. In recent years, their role as efficient blue carbon sinks has been highlighted and estimates have indicated that seagrass meadows contribute up to 15% of the total carbon storage in the ocean [6,9,10]. Impacts of multiple anthropogenic stressors, however, threaten seagrass habitats worldwide [11,12]. Light reduction, primarily caused by eutrophication and sedimentation, is one of the most severe threats [11] as seagrasses have relatively high minimum light requirements for growth [13,14]. Increased nutrient inputs can lead to the mass development of opportunistic macroalgae, resulting in overgrowth and reduced light availability. Suspended matter as a result of dredging or coastal erosion also leads to increased water attenuation [15,16]. Plants can tolerate certain periods of shading by mobilizing stored resources, by adapting to the reduced light by changing the chlorophyll *a* to *b* ratio, or by increasing the concentration of chlorophyll in the leaves [17]. Such adaptations, however, are possible only to a certain extent beyond which total disappearance of the seagrass is expected, and prolonged shading has been demonstrated to have destructive effects on seagrasses [17]. As with light reduction, the impact of overgrazing is well documented [18–21], leading to the denudation of substantial seagrass areas. Possible recovery from overgrazing events depends on species-specific biology, disturbance duration, and the cumulative effects of various stressors.

Seagrasses can store various reserve carbohydrates in their underground root–rhizome systems for mobilization during periods of stress or translocation to damaged shoots to support growth [22]. However, if the stress persists for long periods or becomes chronic, these stored resources are exhausted and the carbon balance of the plant system is altered [22]. This could eventually lead to serious degradation and a cascade of processes that eventually compromise habitat function and plant survival. Several studies have examined the effects of shading and grazing on the health and growth of seagrass habitats in different areas of the world with a focus on different species [17,21] and on the net production of the seagrass systems [23], but little effort has been made to estimate the role of the seagrass plant itself. Also, to capture plant responses at different physiological levels, there is a need to combine biometric methods with in situ productivity estimation that can capture full diel productivity cycles. There is a need to further develop direct non-destructive methods of measuring productivity, such as in situ chlorophyll fluorescence, as an alternative to standard incubation experiments that lead to serious bias in productivity estimation [24].

The overall aim of this study was to assess and understand the effects of shading and simulated grazing on the contribution of seagrass plants to the CO_2_ capture potential of a tropical seagrass meadow (dominated by *Thalassia hemprichii*) after a prolonged period of stress at two intensity levels. In specific, we measured net plant production (based on photosynthetic activity, biomass and morphology), plant growth (based on leaf elongation and lepidochronology) and resource allocation (based on starch and carbohydrate content). We hypothesized that both shading and simulated grazing would negatively affect both the productivity and CO_2_ capture of seagrass habitats, with high-intensity levels resulting in more pronounced impacts. As part of the same larger project Dahl et al [25] discussed the effect of disturbance on sediment carbon storage under the same experimental setting.

## Materials and methods

### Study area and experimental setup

A manipulative field experiment was conducted in Chwaka Bay on the east coast of Unguja Island, Zanzibar (06°09’S, 39°26’E), from November 2013 through March 2014. Chwaka Bay is a semi-enclosed embayment and a “hot spot” for seagrass diversity with up to 11 species of seagrass distributed in monospecific and mixed meadows interspersed with a variety of macroalgae [26]. The bay is characterized by a semi-diurnal tidal regime [27], entpulative experiscase letters mpared to the controlrent leaves of one shoot and between different shoots. and young shoot.sediment of high biogenic origin [28], salinity of 26–35, and water temperatures of 25–35°C during low tide (Dar es Salaam meteorology station).

**Fig 1:**
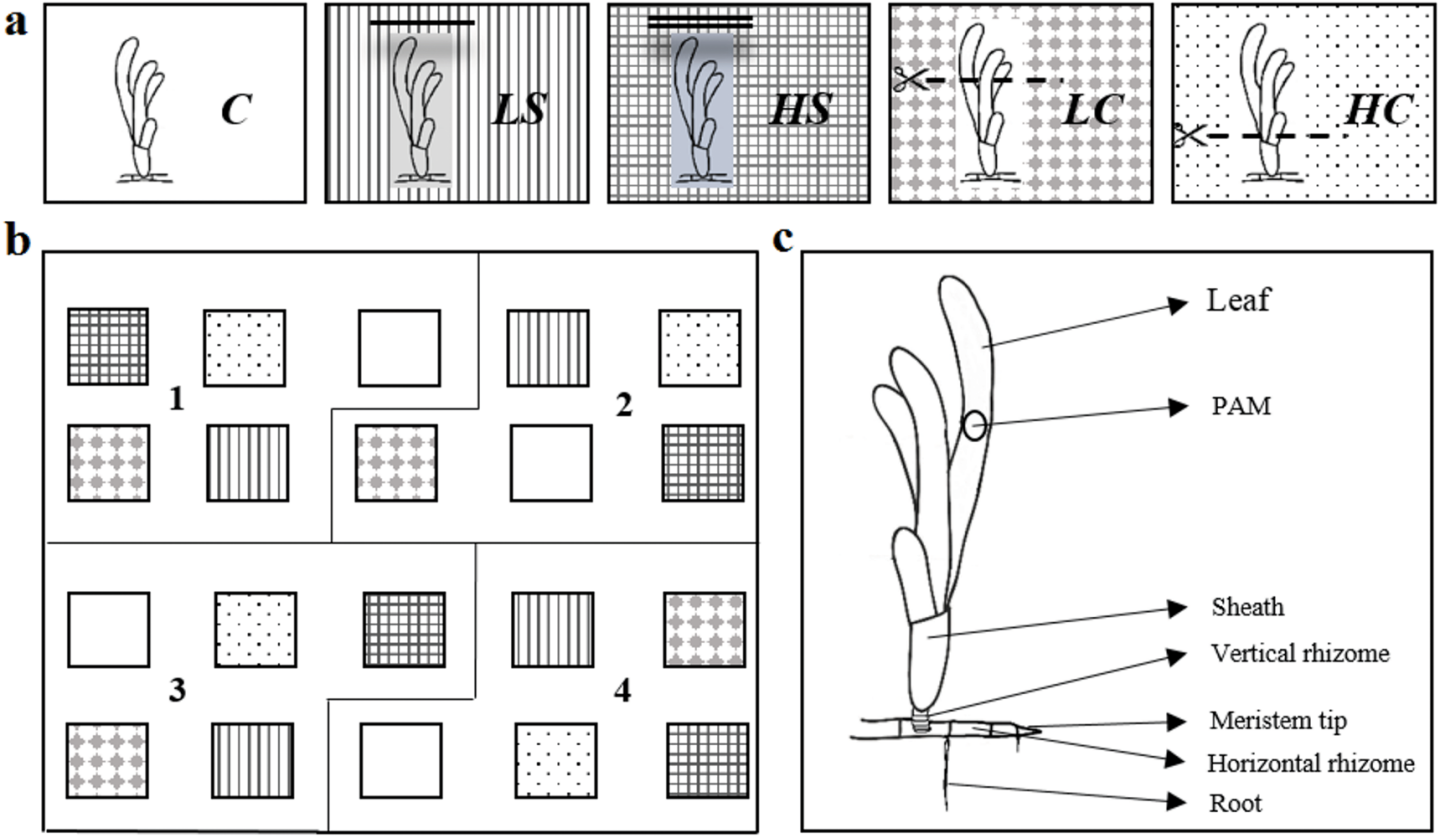
(A) Control, *C*, and treatments: low shading–*LS*, high shading–*HS*, low clipping–*LC*, and high clipping–*HC*. (B) Experimental setting showing randomized complete block design (*n* = 4), with filling patterns corresponding to treatments presented in (A). (C) Schematic of a shoot with rhizomes and roots of a *Thalassia hemprichii* plant. The circle (on the third fully developed leaf) indicates the location where PAM measurements were made.

The experiment was set up in an intertidal area dominated by the climax seagrass *T*. *hemprichii*. Twenty quadrat plots of 10 m^2^ were established and distributed using a randomized complete block design with five treatments, each with four (block) replicates (Fig 1A and 1B). Measurements were made at least 1 m from the plot edge to avoid allocation of resources from nearby seagrass areas [29]. Our experimental design consisted of two intensity levels of shading, two intensity levels of simulated grazing, and untreated seagrass control plots (Fig 1A). Shading screens were mounted about 40 cm above the sediment surface and used to attenuate the light reaching the plants. The mean light attenuation was calculated as a percent reduction from the control plots and was measured to be 64% and 75% of the ambient photosynthetic active radiation (PAR) for the low- (one screen) and high-intensity shading levels (two screens), respectively. Screens were cleaned daily of debris and potential fouling organisms, and replaced twice during the experiment. Grazing was simulated by clipping the seagrass shoots (with scissors) at constant one-week intervals throughout the experiment. The different grazing intensities were simulated by clipping the shoots either to half of their original length or near the leaf sheath for the low- and high-intensity treatments, respectively. All measurements were made at the end of the experiment (mid March) over a three-week period during low tide. The different experimental treatments will subsequently be referred to as LS–low shading, HS–high shading, LC–low clipping, HC–high clipping, and C–control (Fig 1A).

### Biomass and biometric measurements

To determine the above- and belowground dry-weight biomass, shoot density, leaf surface area, and leaf length of the seagrass habitat, three biomass samples were collected from each plot with the aid of a 25 × 25-cm frame. Seagrass shoots were separated into leaf, rhizome, and root fractions, and each plant part was dried at 60°C until constant weight was obtained. All shoots in each sample were counted. To determine the effect of the five-month manipulation on leaf length and width, all plants in the treated plots were left to grow for three weeks before sampling; thereafter, the length of the third fully developed leaf (neither senescent nor immature and unharmed by previous clipping) was used for the measurements.

### Shoot growth

*T*. *hemprichii* is a di-meristematic, leaf-replacing form of seagrass [30] (Fig 1C). This means that it has two types of meristematic regions: one located at the horizontal rhizome tip, responsible for rhizome elongation and the production of new shoots, and one located at the base of the leaf cluster, responsible for leaf elongation and the emergence of new leaves, which are constantly shed and replaced with new ones. To estimate the shoot growth rate and plastochron interval (PI), the puncturing technique [30] was used. The average values of the PI measurements were used in calculating the rhizome elongation rate. In each plot, shoots (*n* = 3–5) were punctured with a needle and after seven days were relocated and harvested for measurements in the laboratory.

### Lepidochronology

To determine whether any changes occurred in plant genet morphology, a reconstructive technique was used. Three genets with a minimum of two intact shoots each were excavated from each plot. Each genet had a rhizome meristem tip, behind which the first and second shoots, called Shoot 1 and Shoot 2, respectively, were used in the measurements. The number and length of rhizome internodes between the meristematic tip of the horizontal rhizome and Shoot 1 and between Shoot 1 and Shoot 2 were measured. The lengths of Shoot 1 and Shoot 2 were also determined.

### Diel fluorescence

Chlorophyll fluorescence is a non-destructive method for measuring photosynthetic efficiency and rates in photosynthesizing organisms [31]. It is a widely used method and has the advantage of permitting the replication of in situ measurements over long periods. The electron transport rates (ETRs) of photosystem II (PSII) can then be used in estimating corresponding O_2_ production or CO_2_ assimilation rates of the targeted plant species [32]. In our experiment, in situ measurements of diel patterns of photosynthesis were made using a classic submersible fluorometer (Aquation, Australia) equipped with five independent sensors and a data logger. Over periods of 24–48 hours, the effective quantum yield of PSII, Φ_PSII_ (i.e., the percent of light energy absorbed by the plant used to drive the photochemical reactions in the photosynthetic apparatus), was recorded simultaneously in five plots (i.e., in one replicate of each treatment) at even intervals of 15 min. For these measurements, the middle section of the third fully grown leaf from a randomly selected shoot was mounted on a clear plastic clip in a vertical position to ensure a fixed distance between the fluorometer and the sample (Fig 1C). A submersible photosynthetic irradiance recording system (Odyssey, Dataflow System, New Zealand) was mounted next to each fluorescence sensor, allowing simultaneous recording of photosynthetically active radiation (PAR, i.e., the light that plants can use in photosynthesis) and fluorescence. The same procedure was repeated for all remaining block replicates over a two-week period. An absorption factor (AF), which accounts for the amount of light absorbed by the leaf tissues, was established for seagrass leaves from each treatment as described in Beer et al. [32]. To calculate the electron transport rate (ETR), the following equation was used:

$$ETR=PAR\times \Phi_{PSII}\times AF\times 0.5$$ (1)

where PAR is the photosynthetically active radiation, *Φ*_PSII_ is the effective quantum yield of PSII, AF is the absorption factor, and 0.5 is the assumed relative distribution of captured photons between PSII and PSI. Seagrass productivity per area and day was calculated as the diel sum of total ETR over 12 h (720 min). As measurements were made every 15 min, ETR (mol e^−^m^–2^ s^–1^) was multiplied by 900 (the number of seconds in 15 min) and all 15-min intervals were summed, as follows:

$$\sum_{i=1}^{48}ETR_{i}\times LA$$ (2)

where ETR_i_ is the i^th^ ETR computed every 15 min over the 12-h period (48 is the number of 15-min intervals in 12 h) and LA is leaf area per m^2^ calculated for each treatment and the control. Specific diel gross productivity (GP_spec_12h) was calculated as:

$$\sum_{i=1}^{48}\frac{{ETR}_{i}\times c}{{AG}_{{DW}^{sp}}}$$ (3)

where *c* = 0.000013203 is a unit transformation constant that includes the ETR to CO_2_ conversion, in which it is assumed that the ETR/O_2_ evolution ratio is 1/4 and the O_2_ evolution/CO_2_ photoassimilation ratio is 1/1.2 [32]; the calculation also includes mole to gram conversions. AG_DW_^sp^ is the specific dry weight of aboveground biomass per leaf area (gDWm^–2^_LA_). Diel gross productivity (GP_12h_) per bottom area (gCO_2_ m^–2^ bottom 24h^-1^) was further calculated as follows:

$${GP}_{12h}={GP}_{spec}12h\times {AG}_{DW}act,$$ (4)

where AG_DW_^act^ is the actual dry weight of aboveground biomass per bottom area (gDW_AG_ m^–2^_bottom_) in each plot at the end of the experiment. No respiration measurements were made during this study; therefore, the productivity/respiration (P/R) ratios for both photosynthetic and non-photosynthetic tissue were taken from the literature in order to calculate diel respiration and gross production. Due to insufficient data on *T*. *hemprichii*, studies of the productivity of both *T*. *hemprichii* and *T*. *testudinum* were used [33–38]. Respiration of the photosynthetic tissue (R_ps_) was calculated as:

$$R_{ps}={ETR}_{mdC}\times c\times \frac{R_{ps}}{NP}lit\times \frac{{AG}_{DW}act}{{AG}_{DW}sp}$$ (5)

where ETR_mdC_ is the mean ETR value obtained every 15 min from the control plots before mid-day (from 10:45 AM to 11:45 AM), c is the unit transformation constant (as above), $\frac{R_{ps}}{NP}lit$ is an R/P ratio for photosynthetic tissue from the literature, and AG_DW_^act^ and AG_DW_^sp^ are as explained above. The end result was recalculated for a 24-hour cycle. The respiration of non-photosynthetic (R_nps_) tissue was calculated as:

$$R_{nps}=R_{ps}\times \frac{R_{nps}}{R_{ps}}lit\times {BG}_{DW},$$ (6)

where $\frac{R_{nps}}{R_{ps}}lit$ is the ratio of the respiration of non-photosynthetic tissue to the respiration of photosynthetic tissue taken from the literature and BG_DW_ is the dry weight biomass of belowground tissue for each treatment. The result was recalculated for a 24-hour cycle. Whole plant respiration was calculated as:

$$R_{plant}=R_{ps}+R_{nps}$$ (7)

Diel net productivity was calculated as:

$${NPP}_{24h}={GP}_{12h}-R_{plant}$$ (8)

As the P/R ratios from the literature differed and the respiration of photosynthetic and non-photosynthetic tissue was not always measured on the same plants, all possible combinations of P/R values from the literature were used in calculating R_ps_ and R_nps_. Individual P/R ratios were calculated for all experimental treatments.

When analysing the fluorescence data, a desiccation effect was observed. This occurred during low tide when the clipped leaf samples were exposed to air. The effect was further analysed by comparing the recovery of Φ_PSII_ after desiccation with measurements recorded at night.

When exposing the plants to increasing light intensities, the corresponding ETR responses can be presented as so-called rapid light curves (RLCs). Such curves are used to convey information about the performance of the plant in different light conditions as well as to estimate the maximum photosynthetic capacity (ETR_max)_, the amount of light at which the photosynthetic apparatus is saturated (E_k_), and photosynthetic efficiency (i.e., the slope of the curve, alpha α) as well as to obtain information about long-term adaptations to different light conditions. In this experiment, RLCs were determined in situ using a WALZ PAM fluorometer (*n* = 6 in each plot). The outcome was fitted to a regression curve after Jassby and Platt [39] using SigmaPlot. The maximum ETR (ETR_max_), saturating irradiance (E_k_), and initial slope of the RLC (α) were derived from the fitting, and compared between control and disturbed treatments.

### Carbohydrates and starch

Biomass samples were collected in each plot for total soluble carbohydrate (TSC) and starch analyses at the end of the experiment. The samples were collected at least 40 cm inward from the perimeter of the plot to avoid margin effects. The mature leaves were quickly rinsed in freshwater and scraped of epiphytes before analysis. Leaves, rhizomes, and roots were sorted, rinsed, and dried at 60°C until constant weight. The dried subsamples from all fractions were later ground into fine powder. TSC was extracted by hydrolysing samples in a boiling water bath for three hours with 5 mL of 2.5N HCl and analysed spectrophotometrically using an anthrone assay standardized to glucose [40]. Starch from ground samples of leaves, rhizomes, and roots was repeatedly extracted in hot 80% ethanol and solubilized in 52% perchloric acid. The green concentrate was subsequently analysed spectrophotometrically (UV-1601-VIS; Shimadzu, Japan) at 630 nm using an anthrone assay [40,41].

### Statistical analysis

One-way or two-way ANOVAs were performed to test for significant differences between the experimental treatments for all response variables. Before the analyses, the assumption of homogeneity of variance was checked using Levene’s [42] test, and data were log10(*x* + 1) or square root transformed when necessary. A posteriori multiple comparison tests were performed using the Student–Newman–Keuls (SNK) procedure. All statistical analyses were conducted using Statistica v. 5.5.

## Results

### Biomass and biometrics

**Fig 2:**
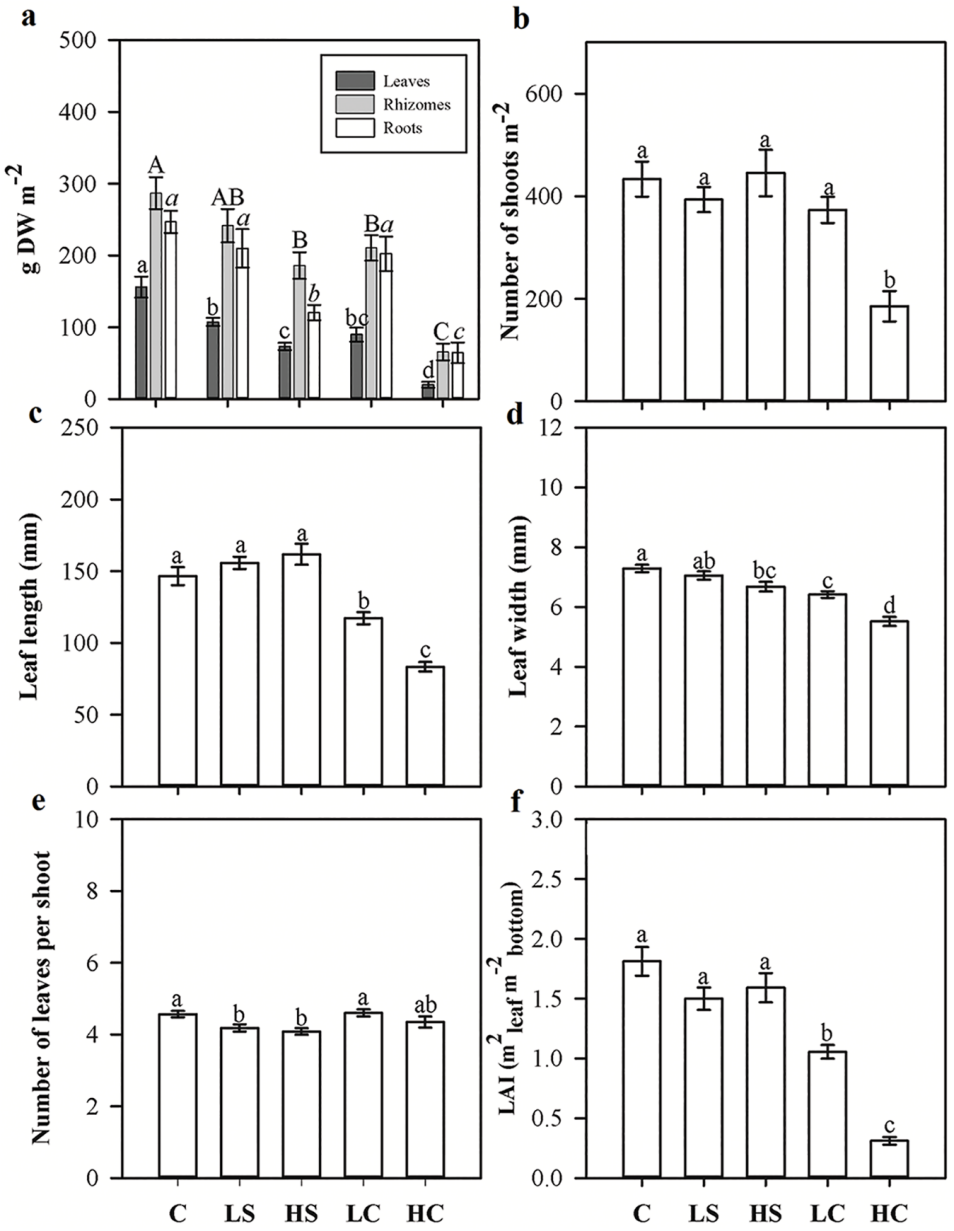
(A) Dry biomass, (B) number of shoots, (C) leaf length, and (D) leaf width measured in treatment and control plots at the end of the four-month manipulative experiment. Measures are mean ±SE, and lower-case letters above bars indicate treatments separated by Student–Newman–Keuls post hoc analyses (*p* < 0.05). For abbreviations, see Fig 1.

The five-month manipulation experiment led to a significant reduction of leaf biomass in all treated plots versus the control plots (Fig 2A). The greatest reduction, >50%, in the biomasses of rhizomes and roots occurred in the HC treatment. Rhizome and root biomasses in the HS treatment as well as the rhizome biomass in the LC treatment were also significantly below the control levels (Fig 2A). The above- to belowground biomass ratio was reduced in the HC treatment compared all other treatments and the control (SNK test, *p* < 0.05). Shoot density was significantly reduced only in the HC treatment (about 50% less than in the control plots), whereas changes in the other treatments were not significant (Fig 2B). Leaf length of the third fully developed leaf was negatively affected by the LC and HC treatments (Fig 2C), and leaf width was significantly reduced in the HS, LC, and HC treatments (Fig 2D). The number of leaves per shoot was reduced in both shading treatments versus the control, but did not differ from the control in the clipping treatments (Fig 2E). The Leaf Area Index (LAI) was clearly lower in both clipping treatments than in the control, while no effects were seen in the shading treatments (Fig 2F).

### Shoot growth

**Fig 3:**
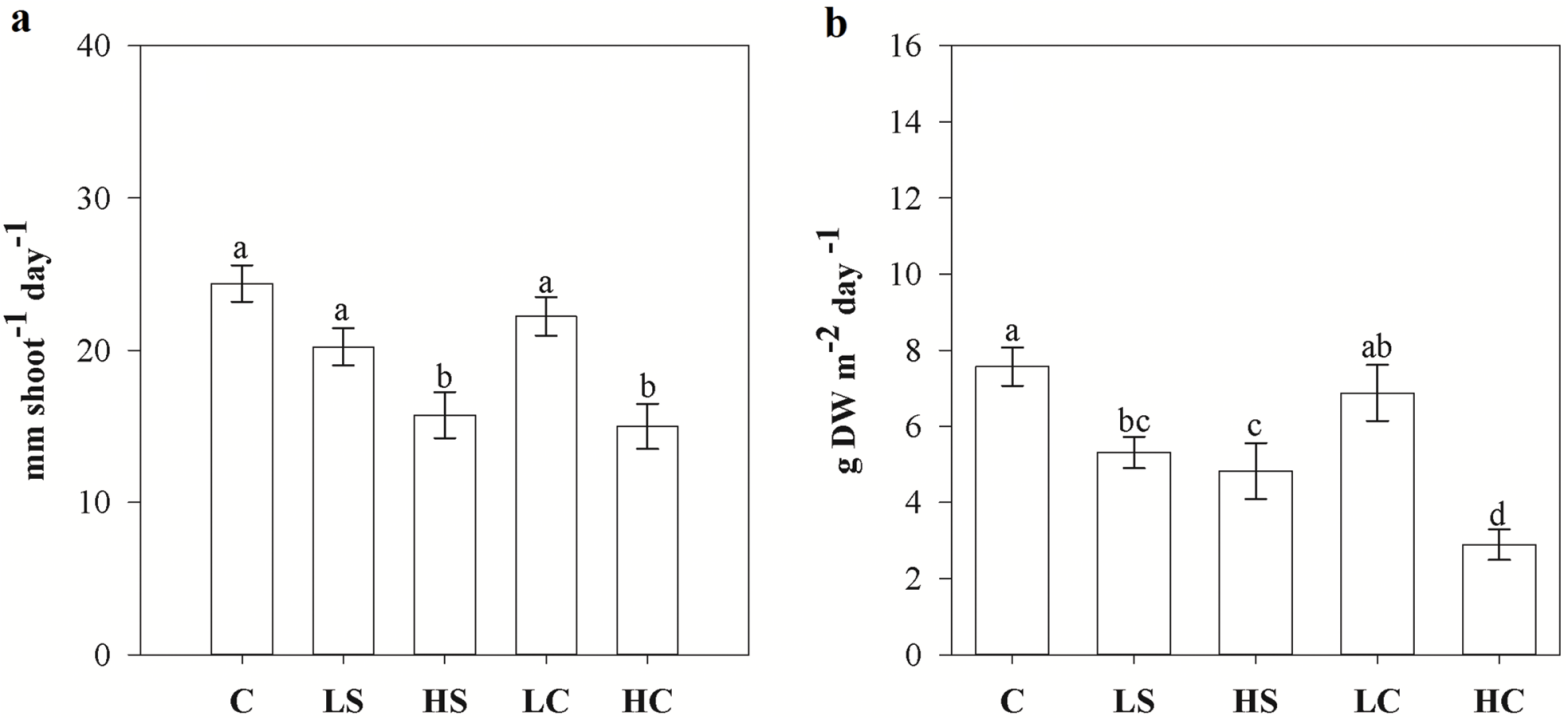
Leaf elongation rate (A) and resulting calculated biomass growth (B) in treatment and control plots at the end of the four-month manipulative experiment. Measures are mean ±SE, and lower-case letters above bars indicate treatments separated by Student–Newman–Keuls post hoc analyses (p < 0.05). For abbreviations, see Fig 1.

The leaf elongation rate was significantly reduced in the HS and HC treatments versus the control (Fig 3A). The leaf biomass growth was significantly reduced in the LS, HS, and HC treatments versus the control (Fig 3B).

### Lepidochronology

**Fig 4:**
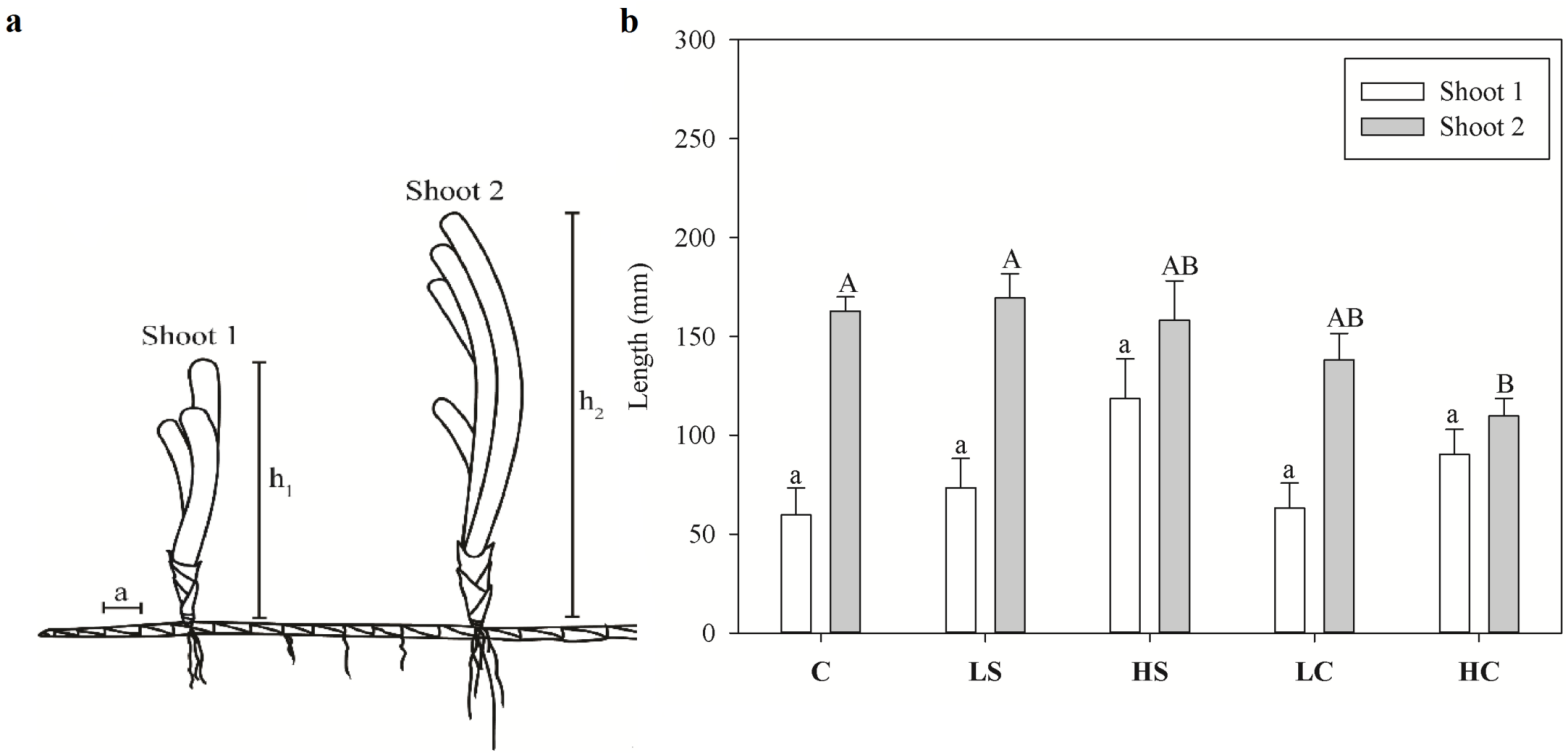
(A) Schematic of growing genet with two intact ramets (shoots) of the seagrass *Thalassia hemprichii*: a–length of an internode segment of the horizontal rhizome; h_1_ and h_2_ –lengths of Shoot 1 and Shoot 2. (B) Length of Shoot 1 (younger) and Shoot 2 (older) left to grow for the duration of the elongation rate experiment. Measures are mean ±SE, and lower-case letters (Shoot 1) and upper-case letters (Shoot 2) above bars indicate treatments separated by Student–Newman–Keuls post hoc analyses (*p* < 0.05). For abbreviations, see Fig 1. Comparison is made between treatments and control within Shoot 1 and Shoot 2.

Reconstruction of early shoot and rhizome growth (Fig 4A) revealed the effects of the treatments on plant development over the experimental period. The average height of Shoot 1 increased in the HS treatment, but not significantly, whereas Shoot 2 was significantly shorter in the HC treatment (Fig 4B). Internode length differed among the treatments, while the number of internodes did not (Table 1). In terms of shoot age, both internode length and number differed clearly between shoots 1 and 2 (Table 1). There was an interaction between treatment and shoot age for internode length (Table 1). The length of the horizontal rhizome internodes between the meristematic tip and Shoot 1 was significantly reduced in all treatments versus the control (SNK test, *p* < 0.05). A similar effect could be seen for the internodes between shoots 1 and 2 only in the HS and HC treatments (SNK test, *p* < 0.05). Between Shoot 1 and the meristematic tip, the number of internodes was higher in the HC than in all other treatments or the control, although the difference from the control was not significant.

**Table 1:** Results of two-way ANOVAs testing the effects of shoot age and treatment on internode length and internode number. Significant values (*p* < 0.05) are shown in bold.

Effect	Internode length			Internode number		
	*df*	MS	p	*df*	MS	p
Shoot	4	4.259	**< 0.001**	4	118.79	0.039
Treatment	1	3.487	**< 0.001**	1	1399.21	**< 0.001**
Treatment*Shoot	4	0.441	**< 0.001**	4	101.71	0.070
Error	1287	0.035		116	45.55	

### Diel productivity

**Fig 5:**
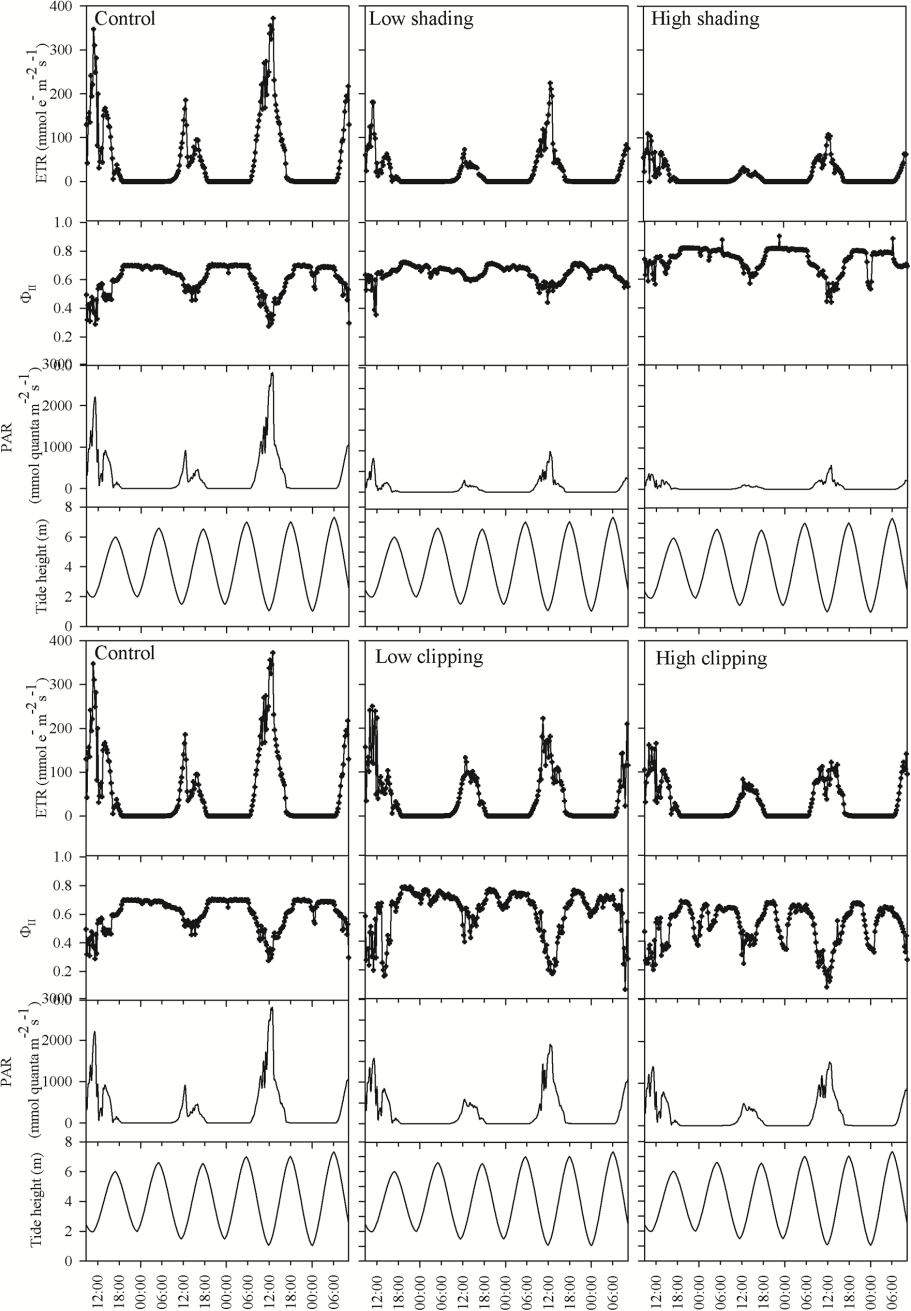
Diel fluctuations in tide height, PAR, ΦII, and ETR for all experimental treatments (i.e., control, low shading, high shading, low clipping, and high clipping). Measurements were made at the end of the five-month manipulative experiment, and the graph shows a representative replicate. Results for the control are shown twice for easier comparison with the treatments.

**Fig 6:**
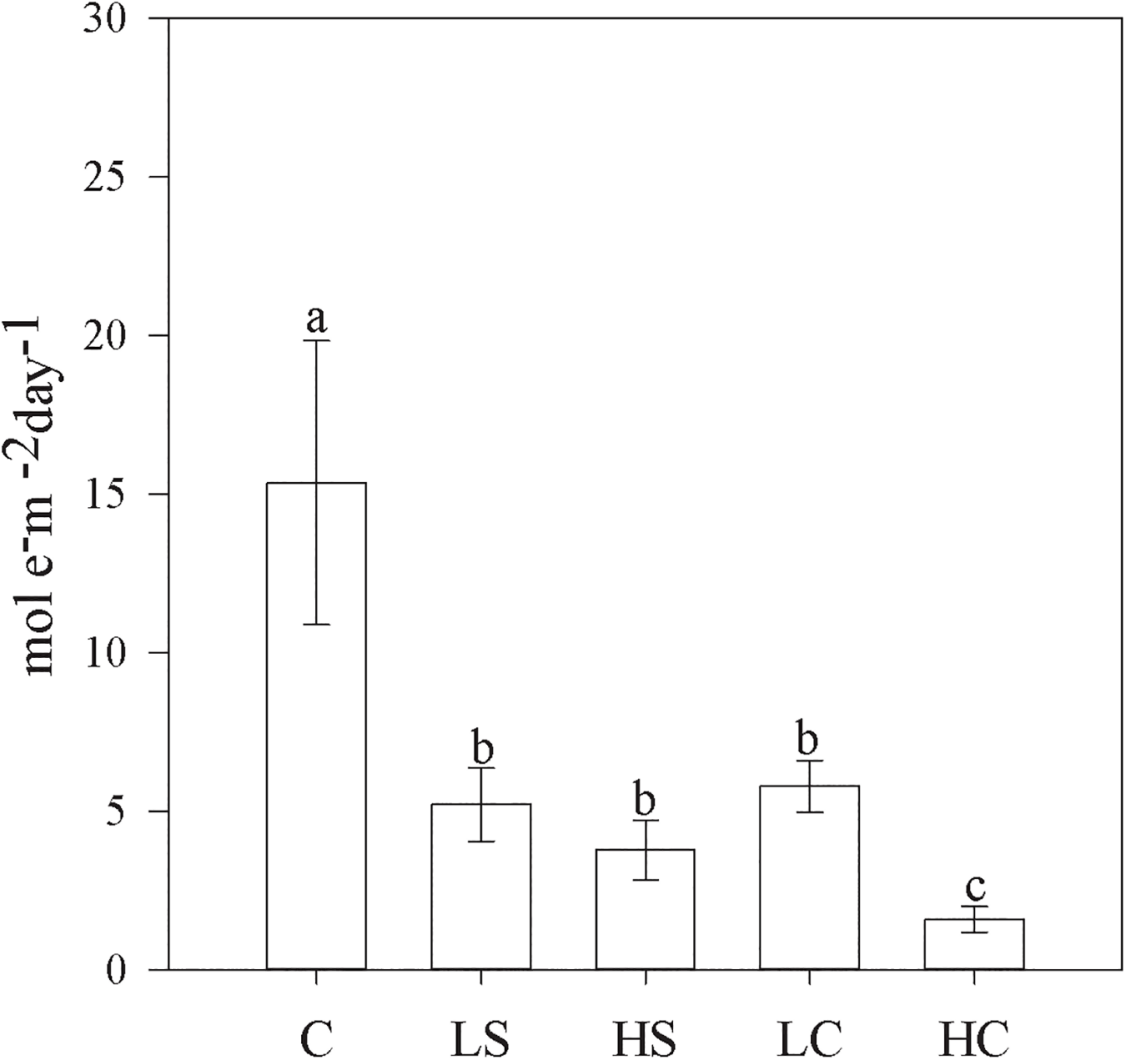
Seagrass photosynthesis per area and day. Measures are mean ±SE, and lower-case letters above bars indicate treatments separated by Student–Newman–Keuls post hoc analyses (*p* < 0.05). For abbreviations, see Fig 1.

**Fig 7:**
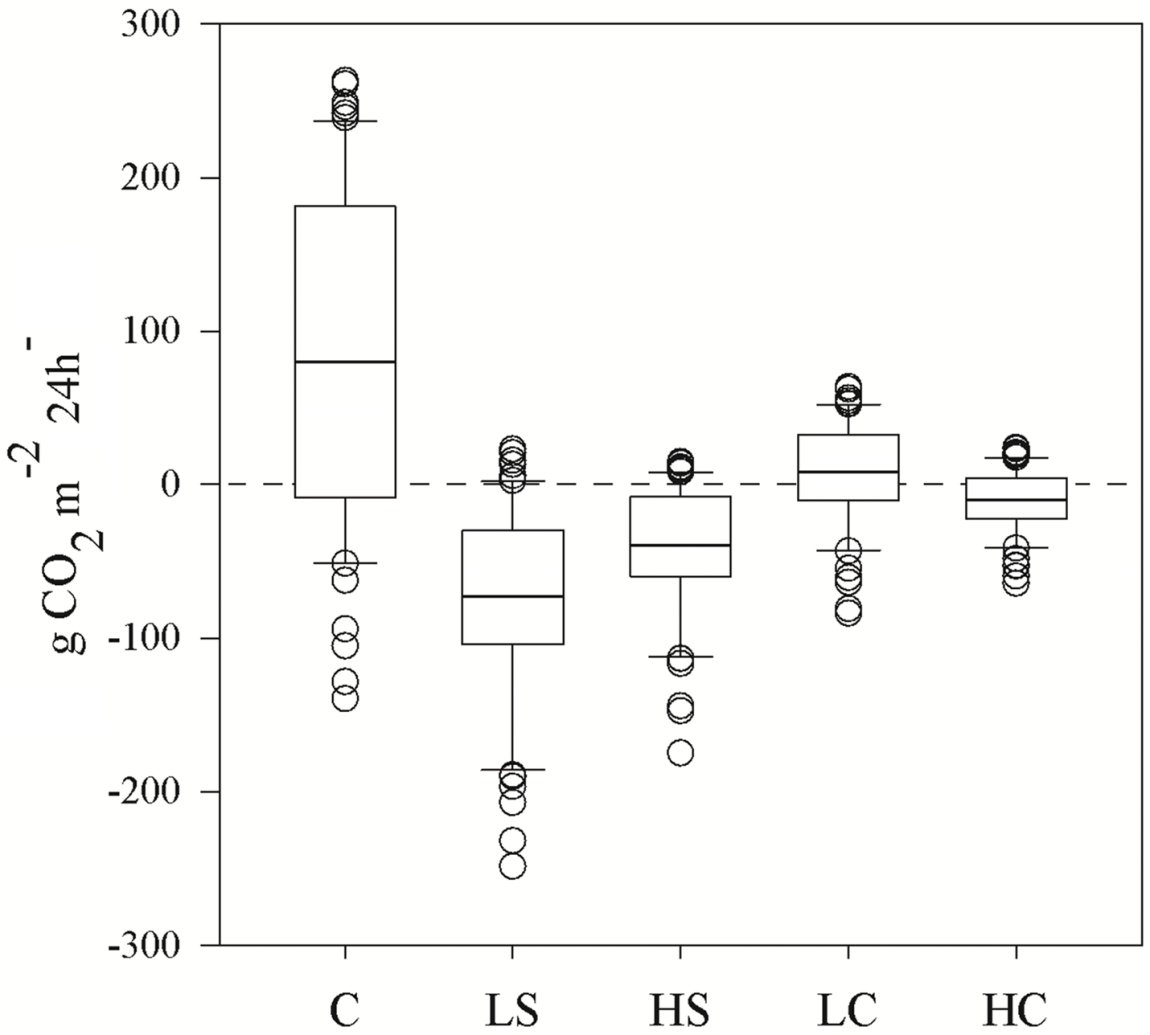
Calculated diel net productivity as CO_2_ fixation per meadow area and day, shown as a range of minimum and maximum values (for the calculations, see “Materials and methods”). In the boxes, lines in middle indicate means, upper and lower lines indicate the 75 and 25 quartiles, respectively, upper and lower whiskers indicate maximum and minimum values, respectively, and circles indicate outliers. Lower-case letters above bars indicate treatments separated by Student–Newman–Keuls post hoc analyses (*p* < 0.05). For abbreviations, see Fig 1.

Diel fluctuations in tidal height, PAR, Φ_PSII_, and ETR are illustrated by a single representative replicate (Fig 5). Changes in Φ_PSII_ and ETR followed the fluctuations in light intensity, although a significant reduction in Φ_PSII_ could be observed during the lowest tidal regime, both in daytime and at night, likely due to the effect of desiccation when the plants became exposed [43]. The recovery of photosynthetic capacity, measured as Fv/Fm, after a desiccation event was not significantly different between the control and any of the disturbed treatments, although the HS and HC treatments displayed slight variation of 90–97% and 90–95%, respectively. The seagrass net productivity per area and day (mol e^−^m^–2^ day^–1^) was reduced by more than 62% in all disturbed treatments versus the control, and was reduced by 89% in the HC treatment (Fig 6). Calculated P/R ratios for the whole plant, photosynthetic tissue, and non-photosynthetic tissue as well as specific diel gross productivity, P (g CO_2_ g DW^–1^), estimates for all treatments are shown in Table 2. Comparing estimated diel net productivity per bottom area (gCO_2_ m^–2^ bottom 24h^-1^) clearly indicates lower values for all disturbed treatments versus the control (ANOVA, *p* < 0.05), with both shading treatments and the HC treatment resulting in negative mean carbon fixation values over a 24-hour cycle (Fig 7).

**Table 2:** Results of one-way ANOVA testing differences between control and all treatments in maximum electron transport rate (ETR_max_, μmol e^−^m^–2^ s–1), initial slope of the RLC (α) and saturation irradiance (*E*_k,_ μmol_photons_ m^–2^ s^–1^)_,_ productivity (P, gCO_2_ gDW^–1^), and P/R ratios for the whole plant (P/R_plant_), photosynthetic tissue (P/R_ps_), and non-photosynthetic tissue (P/R_nps_). Post hoc analyses are performed using the Student–Newman–Keuls method. Significant values (*p* < 0.05) are shown in bold.

Treatment	ETR _max_	p	α	p	*E*_k_	p	P*	P/R _plant_*	P/R _ps_*	P/R _nps_*
	MEAN±SE		MEAN±SE		MEAN±SE		MEAN±SE			
C	24.23 ± 14.4		0.13 ± 0.04		192.79 ± 92.6		1.21 ± 0.354	2.19	3.30	6.51
LS	18.14 ± 11.6	0.146	0.10 ± 0.02	**0.036**	172.31 ± 81.0	0.221	0.60 ± 0.132	0.77	1.34	1.79
HS	15.41 ± 7.2	**0.015**	0.10 ± 0.02	**0.006**	155.90 ± 45.8	0.052	0.35 ± 0.088	0.42	0.77	0.93
LC	21.80 ± 5.8	0.833	0.12 ± 0.02	0.954	187.61 ± 46.7	0.916	0.78 ± 0.111	1.52	2.63	3.58
HC	28.25 ± 12.9	0.467	0.16 ± 0.07	**0.023**	186.49 ± 72.9	0.991	1.11 ± 0.285	0.97	2.10	1.81

* Data recalculated from ETR values using conversion factors from the literature (see “Materials and methods”).

Parameters derived from the RLCs are shown in Table 2. ETR_max_ was significantly lower in the HS treatment than the control (Table 2). The initial slope of the RLCα for both the LS and HS treatments was significantly lower than that of the control, whereas that of the HC treatment was significantly higher (Table 2). E_k_ did not differ significantly between treatments and the control (Table 2).

### Total soluble carbohydrates and starch

**Fig 8:**
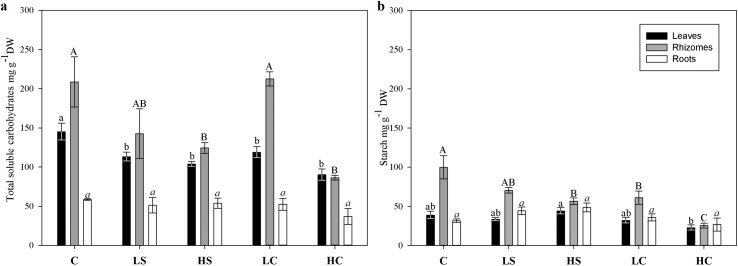
(A) Total soluble sugar content and (B) starch in leaves, rhizomes, and roots. Measures are mean ±SE, and letters above bars indicate treatments separated by Student–Newman–Keuls post hoc analyses (*p* < 0.05). For abbreviations, see Fig 1.

The TSC concentration in the leaves was significantly affected by all disturbed treatments to a somewhat similar degree compared with the control (Fig 8A). In rhizomes, significant depletion of TSC could be seen both in the HS and HC treatments, the latter causing more than a 50% decline, whereas the TSC concentration in roots was unaffected by any disturbed treatment (Fig 8A). The starch content was significantly reduced only in the rhizomes: the HC treatment had a drastic effect, causing almost a 75% decline in starch concentration, the HS and LC treatments caused a moderate decline, whereas the LS treatment had no effect (Fig 8B).

## Discussion

This study has illustrated how prolonged shading and simulated grazing resulted in an overall decline in seagrass productivity, production, and stored carbohydrates, leading to a drastically lower areal productivity that will consequently reduce the carbon sink capacity of the seagrass meadow. Our results are based on measurements of the impacts of the stress factors on physiological and morphological characteristics of the plants and on estimated respiration rates based on literature data. We found that a substantial reduction in the measured parameters, i.e., biomass, biometrics, growth, photosynthetic performance, and stored reserves, occurred after five months, resulting in a negative carbon balance and reduced total daily (net and gross) productivity of *T*. *hemprichii* in all disturbed treatments. Morphological changes such as reduced leaf size, leaf number, and shoot density were of high importance for the observed loss in areal productivity. Decreased growth rates, depleted carbohydrate reserves, and reduced photosynthetic performance further illustrate the negative effects of these disturbances on the seagrass. Shading reduced the available energy for production, while clipping directly reduced the amount of green tissue responsible for plant productivity. Although different stressors generally affect plants in specific ways, we found that both shading and simulated grazing had similar consequences for overall plant production and for the seagrasses’ capacity to sequester carbon.

### Physiological adaptations

Adaptation to stress involves various mechanisms depending on the intensity, type, and duration of disturbance. Physiological adaptations of the photosynthetic apparatus are commonly seen when plants are exposed to reduced light conditions [32]. To increase productivity under such conditions, plants need to invest energy in a more effective photosynthetic apparatus. Chlorophyll concentrations then increase and the plants will better utilize the reduced light [44]. In our study, such adaptation was seen as a significant decrease in the alpha slope of the light curve in the shading treatments versus the control. No significant effect on the saturating irradiance (E_k_) was observed, however, although a strong decreasing trend was seen in the HS treatment. Additionally, both maximum ETR and specific diel gross productivity (g CO_2_ g DW^–1^) were significantly reduced in the HS treatment and a trend towards reduction was seen in the LS treatment. In contrast to plant responses to shading, grazed plants normally adapt by increasing the photosynthetic activity of the remaining tissue immediately after grazing in compensation and to stimulate regrowth [45,46]. We found no significant change in photosynthetic performance in the LC treatment versus the control, but we did find significantly higher photosynthetic efficiency in the HC treatment than the control. This suggests an adaptation of the photosynthetic apparatus to compensate for tissue removal. The observed reduction in carbohydrate reserves corresponds closely to the reduction in net productivity. Although there is a significant decrease in starch content in the main storage organ (i.e., the rhizomes), *T*. *hemprichii* seems able to cope with and sustain such prolonged stress rather well.

The studied seagrass *T*. *hemprichii* displays traits of a species able to adapt physiologically to grazing. Such traits are the possession of storage organs, an ability to translocate resources, and the capacity to increase photosynthetic efficiency [47]. These traits are also advantageous when coping with other types of stressors, such as light reduction. Previous studies have observed a temporal sequence of plant reactions in response to light reduction. First, the photosynthetic performance of the plant adapts. Then changes in growth rate and biomass appear as well as changes in plant morphology [48], followed by defoliation and shoot mortality [15,49–51]. The magnitude of these effects depends on the strength of the imposed stress [50]. Grazing, depending on its frequency and intensity, can stimulate and enhance plant growth [52]. One way for the plants to compensate for the effects of stress is by using stored reserves in the belowground organs [53]; however, these reserves are restricted so such compensation cannot be maintained for more than a limited period. The use of stored resources can initially stimulate higher production rates, which will facilitate the investment of energy in developing compensatory morphological features, such as leaf production, a higher growth rate, or larger leaf area, which can in turn help in coping with stress. However, if the effect persists for too long, the resources will be exhausted, as was demonstrated here. Thereby, the plants cannot compensate and instead have to adapt by reducing shoot density and total leaf area [54].

### Biomass changes

During the study, both plant biomass and morphology were somewhat Faffected by all disturbed treatments. The reduction in aboveground biomass observed in the two shading treatments was a combined effect of reduced leaf numbers (defoliation) and the reduced width of remaining leaves, as is obvious in the HS treatment. Intensified leaf shedding as well as reduced leaf width and length have been observed in some species of seagrass in response to shading [15,49,51,55], although the effects may vary considerably [56]. Shading did not cause a significant change in either leaf length or above- to belowground ratio in our study, although such responses have been observed in other seagrass species during reduced light conditions [49,57–59]. These types of morphological responses vary with species, but decreased self-shading as a consequence of the thinning of dense seagrass stands has generally been seen as an adaptation to reduced light conditions [15,49,50,55,57]. When comparing the length of the third mature leaf in each treatment, both clipping treatments resulted in much shorter leaves (i.e., leaves that regrew after the clipping) than in the control. This could be a result of the reduced growth rate, but also could indicate reduced shoot height as an adaptation to the constant clipping and reduction of resources. Leaves of seagrass exposed to clipping were narrower in both intensity-level treatments. Such an effect has previously been observed in *T*. *testudinum* [54] exposed to turtle and fish grazing, while a study of *T*. *hemprichii* found no effect of clipping on leaf width [52]. Contrary to other studies of *T*. *testudinum* [49,51,55], in the present study seagrass shoot density was affected only by the HC treatment, while no effect was seen in the shading treatments. However, this could be yet another species-specific plant response and also an indication of the better resistance of *T*. *hemprichii* to stress.

### Effects on growth and the allocation of resources

Reduction of plant growth was observed in the two shading treatments and in the HC treatment. Shoot-scale effects were visible in the high-intensity treatments only, but when taking into account the morphological changes and scaling up to areal production, the effect of the LS treatment was also significant. Similar to the present study, Alcoverro and Mariani [60] found no significant reduction in the growth of *T*. *hemprichii* after four months of clipping the tops of the leaves (comparable to the LC treatment in the present study), possibly explained by allocation of stored energy resources. The reduction in TSC resources observed here could be the result of the prolonged support of growth by the stored resources. Reduced productivity in shaded green tissue could be compensated for by relocation of stored resources from underground tissue [29,61]. Although the starch content of the rhizomes decreased in the two clipping treatments, the LC treatment had no effect on the TSC content of the rhizomes. Effects on starch are generally species specific and even site or season specific [62] and depend on the ability of the plants to translocate stored reserves and nitrogen from the rhizomes to support and maintain plant growth [29,61]. Several studies have illustrated how stress factors trigger the use of stored reserves in seagrasses [63,64]. The translocation of carbohydrates from neighbouring shoots and the ability to support growth are important advantages of plants, such as seagrasses, with clonal vegetative growth. Species such as *T*. *hemprichii* with large underground biomass can support growth during long periods of stress [61]. For example, increased growth is a known response of plants following grazing [65]. Lepidochronology also revealed the effect of prolonged stress, which was most evident in the HC treatment, where older shoots were generally much shorter than in all other treatments. The younger shoots showed signs of increased compensatory growth in the two shading treatments, probably supported by the remaining carbohydrate reserves in the rhizomes.

### Effects on areal productivity and carbon capture of the plants

Areal photosynthetic activity as presented here is calculated from measured ETR and estimated leaf area for each treatment and thus represents the gross production of the seagrass plants. The results (presented in Fig 6) indicate a drastic reduction in productivity for all the disturbed treatments. In the case of shading, this is obviously because of the reduced light available for photosynthesis, whereas in the clipping treatments, the same result is caused by reduced leaf surface area. Interestingly, different types of disturbances lead to a similar reduction in productivity in these plants, although the reduction in the HC treatment is much more pronounced than in the other disturbed treatments versus the control. The situation changes slightly when respiration estimates are included in the calculation of areal production (Fig 7). We estimated similar values for net CO_2_ fixation of the control to the one presented by Longstaff et al. [66] for *Thalassia hemprichii*. In their study, the net CO_2_ fixation was estimated to be 395,7 nmol O_2_ g^-1^ DW s^-1^, which after recalculation was 140,9 g CO_2_ m^-2^ bottom 24h^-1^. The calculated net CO_2_ fixation of the plants in the shaded plots now became lower than that in the clipped plots, while the efficiency of the plants in the clipped plots was comparable to that of the control plants. This was also demonstrated by the calculated specific diel productivity values (g CO_2_ g DW^–1^), which did not differ from that of the control in either of the two clipping treatments. Such high productivity potential compensates somewhat for the severe tissue removal and, as a result, plants manage to keep their net carbon balance around zero (Fig 7). Lower productivity in response to light reduction made the daily carbon balance negative even though the aboveground tissue biomass was clearly greater than that in the clipped plots.

Our experiment demonstrated that a five-month period of stress had clear negative effects on the carbon capture capacity of the seagrass plants. If the conditions of the high-intensity treatments had been extended, the seagrass plants would probably have died, while at lower treatment intensities, the plants might have adapted physiologically. However, as the productivity would decrease, less carbon would be fixed by the plants and consequently less carbon transferred to the belowground plant parts in the sediment. Reduced canopy height and shoot density will also limit the ability of a seagrass meadow to help capture suspended matter. These adverse effects, combined with the calculated negative carbon balance of the shaded plots, the near neutral balance of the clipped plots, and the fully exhausted carbohydrate reserves, might convert these otherwise carbon-sink habitats into a potential carbon source. Consequently, the carbon capture and storage of seagrass habitats could be severely affected, leading to a reduced blue carbon sink capacity.

## References

[1] Gattuso J-P, Gentili B, Duarte CM, Kleypas JA, Middelburg JJ, Antoine D. Light availability in the coastal ocean: impact on the distribution of benthic photosynthetic organisms and contribution to primary production. Biogeosciences Discuss. 2006;3(4):895–959.

[2] Anthony KRN, Ridd P V., Orpin AR, Larcombe P, Lough J. Temporal variation in light availability in coastal benthic habitats: Effects of clouds, turbidity, and tides. Limnol Oceanogr. 2004;49(6):2201–11.

[3] Harper JL. Population Biology of Plants. London: Academic press; 1977. 892 p.

[4] Valentine JF, Heck KL, Busby J, Webb D. Experimental evidence that herbivory increases shoot density and productivity in a subtropical turtlegrass (*Thalassia testudinum*) meadow. Oecologia. 1997;112(2):193–200. doi: 10.1007/s004420050300 28307570 10.1007/s004420050300

[5] Kelkar N, Arthur R, Marbà N, Alcoverro T. Greener pastures? High-density feeding aggregations of green turtles precipitate species shifts in seagrass meadows. J Ecol. 2013;101(5):1158–68.

[6] Laffoley D, Grimsditch G. The Management of Natural Coastal Carbon Sinks. 2009.

[7] Björk M, Short FT, McLeod E, Beer S, Iucn. Managing Seagrasses for Resilience to Climate Change. IUCN Resilience Science Group Working Paper Series. The International Union for the Conservation of Nature and Natural Resources / The Nature Conservancy; 2008. 56 p.

[8] Costanza R, D’Arge R, de Groot R, Farber S, Grasso M, Hannon B, et al The value of the world’s ecosystem services and natural capital. Nature. 1998;387(6630):253–60.

[9] Duarte CM, Cebrián J. The fate of marine autotrophic production. Limnol Oceanogr. 1996;41(8):1758–66.

[10] McRoy C, McMillan C. Production ecology and physiology of seagrasses In: McRoy C, Helfferich C, editors. Seagrass Ecosystems. New York: Marcel Dekker; 1977 p. pp 53–87.

[11] Orth RJ, Carruthers TJB, Dennison WC, Duarte CM, Fourqurean JW, Heck KL, et al A Global Crisis for Seagrass Ecosystems. Bioscience. 2006;56(12):987.

[12] Waycott M, Duarte CM, Carruthers TJB, Orth RJ, Dennison WC, Olyarnik S, et al Accelerating loss of seagrasses across the globe threatens coastal ecosystems. Proc Natl Acad Sci USA. 2009;106(30):12377–81. doi: 10.1073/pnas.0905620106 19587236 10.1073/pnas.0905620106 PMC2707273

[13] Cayabyab NM, Enríquez S. Leaf photoacclimatory responses of the tropical seagrass Thalassia testudinum under mesocosm conditions: a mechanistic scaling-up study. New Phytol. 2007;176(1):108–23. doi: 10.1111/j.1469-8137.2007.02147.x 17696981 10.1111/j.1469-8137.2007.02147.x

[14] Dennison WC, Orth RJ, Moore K a, Stevenson JC, Carter V, Kollar S, et al Assessing Water Quality with Submersed Aquatic Vegetation. Bioscience. 1993;43(2):86.

[15] Ruiz JM, Romero J. Effects of disturbances caused by coastal constructions on spatial structure, growth dynamics and photosynthesis of the seagrass *Posidonia oceanica*. Mar Pollut Bull. 2003;46(12):1523–33. doi: 10.1016/j.marpolbul.2003.08.021 14643778 10.1016/j.marpolbul.2003.08.021

[16] Short FT, Wyllie-Echeverria S. Natural and human-induced disturbance of seagrasses. Environ Conserv. 1996;23(1):17.

[17] Ralph PJ, Durako MJ, Enríquez S, Collier CJ, Doblin M a. Impact of light limitation on seagrasses. J Exp Mar Bio Ecol. 2007;350(1–2):176–93.

[18] Camp DK, Cobb SP, Van Breedveld JF. Overgrazing of Seagrasses by a Regular Urchin, *Lytechinus variegatus*. American institute of Biological Sciences 1973.

[19] Rose CD, Sharp WC, Kenworthy WJ, Hunt JH, Lyons WG, Prager EJ, et al Overgrazing of a large seagrass bed by the sea urchin *Lytechinus variegatus* in Outer Florida Bay. Mar Ecol Prog Ser. 1999;190:211–22.

[20] Peterson BJ, Rose CD, Rutten LM, Fourqurean JW. Disturbance and recovery following catastrophic grazing: studies of a successional chronosequence in a seagrass bed. Oikos. 2002;97(3):361–70.

[21] Eklöf JS, de la Torre-Castro M, Gullström M, Uku J, Muthiga N, Lyimo T, et al Sea urchin overgrazing of seagrasses: A review of current knowledge on causes, consequences, and management. Estuar Coast Shelf Sci. 2008;79(4):569–80.

[22] Larkum AWD, Orth R., Duarte C. Seagrasses: Biology, Ecology and Conservation. Dordrecht: Springer; 2006. 691 p.

[23] Duarte CM, Marba N, Gacia E, Fourqurean JW, Beggins J, Barron C, et al Seagrass community metabolism: Assessing the carbon sink capacity of seagrass meadows. Global Biogeochem Cycles. 2010;24(4):1–8.

[24] Olivé I, Silva J, Costa MM, Santos R. Estimating Seagrass Community Metabolism Using Benthic Chambers: The Effect of Incubation Time. Estuaries and Coasts. 2016;39(1):138–44.

[25] Dahl M, Deyanova D, Lyimo LD, Näslund J, Samuelsson GS, Mtolera MSP, et al Effects of shading and simulated grazing on carbon sequestration in a tropical seagrass meadow. J Ecol. 2016;104(3):654–64.

[26] Gullström M, Lundén B, Bodin M, Kangwe J, Öhman MC, Mtolera MSP, et al Assessment of changes in the seagrass-dominated submerged vegetation of tropical Chwaka Bay (Zanzibar) using satellite remote sensing. Estuar Coast Shelf Sci. 2006;67(3):399–408.

[27] Cederlöf U, Rydberg L, Mgendi M, Mwaipopo O. Tidal Exchange in a Warm Tropical Lagoon: Chwaka Bay. Ambio. 1995;24:458–64.

[28] Mussaka ANN, Kangwe JW, Nyandwi N, Wannäs KO, Mtolera MSP, M. B. Preliminary results of sediment sources, grain size distribution and percentage cover of sand producing Halimeda species and associated flora in Chwaka bay, Tanzania. In: 20th Anniversary Conference on Advances on Marine Sciences in Tanzania 28 June- 1 July 1999, Zanzibar (Tanzania), IMS/WIOMSA. 1999. p. 569.

[29] Marba, Hemminga MA, Mateo MA, Duarte CM, Mass YEM, Terrados J, et al Carbon and nitrogen translocation between seagrass ramets. Mar Ecol Prog Ser. 2002;226:287–300.

[30] Short FT, Duarte CM. Methods for the measurement of seagrass growth and production In: Short FT, Coles RG, editors. Gobal Seagrass Research Methods. Amsterdam: ELSEVIER; 2001 p. 155–82.

[31] Beer S, Björk M, Gademann R, Ralph P. Measurements of photosynthetic rates in seagrasses In: Short FT, Coles RG, editors. Global Seagrass Research Methods. Elsevier Sicience; 2001 p. 183–98.

[32] Beer S, Björk M, Beardall J. Photosynthesis in the Marine Environment. Second Wiley Balckwell; 2014. 224 p.

[33] Herzka SZ, Dunton KH. Seasonal photosynthetic patterns of the seagrass *Thalassia testudinum* in the western Gulf of Mexico. Mar Ecol Prog Ser. 1997;152(1–3):103–17.

[34] Fourqurean JW, Zieman JC. Photosynthesis, respiration and whole plant carbon budget of the seagrass Thalassia testudinum. Mar Ecol Prog Ser. 1991;69(1–2):161–70.

[35] Buesa RJ. Photosynthesis and Respiration of Some Tropical Marine Plants. Aquat Bot. 1977;3:203–216.

[36] Hena MKA, Misri K, Sidik BJ, Hishamuddin O, Hidir H. Photosynthetic and Respiration Responses of Dugong Grass *Thalassia hemprichii* (Ehrenb) Aschers. at Teluk Kemang Seagrass Bed, Malaysia. Pakistan J Biol Sci. 2001;4(12):1487–9.

[37] Agawin NSR, Duarte CM, Fortes MD, Uri JS, Vermaat JE. Temporal changes in the abundance, leaf growth and photosynthesis of three co-occurring Philippine seagrasses. J Exp Mar Bio Ecol. 2001;260(2):217–39. 11358580 10.1016/s0022-0981(01)00253-2

[38] Qasim S, Bhattathiri PMA, Reddy CVG. Primary production of an atoll in the Laccadives. Int Rev ges Hydrobiol. 1972;57(2):207–25.

[39] Jassby AD, Platt T. Mathematical formulation of the relationship between photosynthesis and light for phytoplankton. Limnol Oceanogr. 1976;21(July):540–7.

[40] Hedge JE, Hofreiter BT. Carbohydrate chemistry 17. Whistler RL, Be Miller JN, editors. New York: Academic press; 1962.

[41] Thayumanavan B, Sadasivan S. Physiocochemical basis for the preferential uses of certain rice varieties. Plant Foods Hum Nutr. 1984;34:253–9.

[42] Levene H. Robust Tests for Equality of Variances In: Olkin I, Ghurye S, Hoeffding W, Madow W, Mann H, editors. Contributions to Probability and Statistics: Essays in Honor of Harold Hotelling. Stanford, Calif: Stanford University Press; 1960 p. 278–92.

[43] Björk M, Uku J, Weil A, Beer S. Photosynthetic tolerances to desiccation of tropical intertidal seagrasses. Mar Ecol Prog Ser. 1999;191:121–6.

[44] Silva J, Barrote I, Costa MM, Albano S, Santos R. Physiological responses of *Zostera marina* and *Cymodocea nodosa* to light-limitation stress. PLoS One. 2013 1;8(11):e81058 doi: 10.1371/journal.pone.0081058 24312260 10.1371/journal.pone.0081058 PMC3842938

[45] Briske D, Richards J. Plant responses to defoliation: A physiological, morphological and demographic evaluation. Wild plants Physiol Ecol Dev Morphol. 1995;635–710.

[46] Vergés A, Pérez M, Alcoverro T, Romero J. Compensation and resistance to herbivory in seagrasses: Induced responses to simulated consumption by fish. Oecologia. 2008;155(4):751–60. doi: 10.1007/s00442-007-0943-4 18193292 10.1007/s00442-007-0943-4

[47] Belsky AJ, Carson WP, Jensen CL, Fox GA. Overcompensation by plants:herbivore optimization or red herring? Evol Ecol. 1993;7(1):109–21.

[48] Ruiz JM, Romero J. Effects of in situ experimental shading on the Mediterranean seagrass *Posidonia oceanica*. Mar Ecol Prog Ser. 2001;215:107–20.

[49] Carlson PR, Acker JC. Effects of in situ shading on Thalassia testudinum: preliminary experiments. In: Webb FJ, editor. 12th Ann Conf Wetland Restoration Creation. Tampa, Florida; 1985. p. 64–73.

[50] Collier CJ, Lavery PS, Ralph PJ, Masini RJ. Shade-induced response and recovery of the seagrass *Posidonia sinuosa*. J Exp Mar Bio Ecol. Elsevier B.V.; 2009;370(1–2):89–103.

[51] Czerny AB, Dunton KH. The Effects of in situ Light Reduction on the Growth of Two Subtropical Seagrasses, *Thalassia testudinum* and *Halodule wrightii*. Estuaries. 1995;18(2):418.

[52] Eklöf JS, Gullström M, Björk M, Asplund ME, Hammar L, Dahlgren a., et al The importance of grazing intensity and frequency for physiological responses of the tropical seagrass *Thalassia hemprichii*. Aquat Bot. 2008;89(3):337–40.

[53] Suzuki J, Stuefer J. On the ecological and evolutionary significance of storage in clonal plants. Plant Species Biol. 1999;11–7.

[54] Lacey EA, Collado-Vides L, Fourqurean JW. Morphological and physiological responses of seagrasses (Alismatales) to grazers (Testudines: Cheloniidae) and the role of these responses as grazing patch abandonment cues. Rev Biol Trop. 2014;62(4):1535–48. 25720186 10.15517/rbt.v62i4.12844

[55] Lee KS, Dunton KH. Effects of in situ light reduction on the maintenance, growth and partitioning of carbon resources in *Thalassia testudinum* Banks ex Konig. J Exp Mar Bio Ecol. 1997;210(1):53–73.

[56] Collier CJ, Waycott M, Ospina AG. Responses of four Indo-West Pacific seagrass species to shading. Mar Pollut Bull. Elsevier Ltd; 2012;65(4–9):342–54. doi: 10.1016/j.marpolbul.2011.06.017 21741666 10.1016/j.marpolbul.2011.06.017

[57] Gordon DM, Grey KA, Chase SC, Simpson CJ. Changes to the structure and productivity of a *Posidonia sinuosa* meadow during and after imposed shading. Aquat Bot. 1994;47(3–4):265–75.

[58] Hemminga MA. The root/rhizome system of seagrasses: an asset and a burden. J Sea Res. 19980;39(3–4):183–96.

[59] Longstaff B., Loneragan N., O’Donohue M., Dennison W. Effects of light deprivation on the survival and recovery of the seagrass *Halophila ovalis* (R.Br.) Hook. J Exp Mar Bio Ecol. 1999;234(1):1–27.

[60] Alcoverro T, Mariani S. Patterns of fish and sea urchin grazing on tropical Indo-Pacific seagrass beds. Ecography (Cop). 2004;27(3):361–5.

[61] Marbà N, Hemminga MA, Duarte CM. Resource translocation within seagrass clones: Allometric scaling to plant size and productivity. Oecologia. 2006;150(3):362–72. doi: 10.1007/s00442-006-0524-y 16944245 10.1007/s00442-006-0524-y

[62] Cebrian J, Duarte CM, Agawin NSR, Merino M. Leaf growth response to simulated herbivory: a comparison among seagrass species. J Exp Mar Bio Ecol. 1998;220:67–81.

[63] Collier CJ, Prado P, Lavery PS. Carbon and nitrogen translocation in response to shading of the seagrass *Posidonia sinuosa*. Aquat Bot. 2010;93(1):47–54.

[64] Nayar S, Collings GJ, Miller DJ, Bryars S, Cheshire AC. Uptake and resource allocation of inorganic carbon by the temperate seagrasses *Posidonia* and *Amphibolis*. J Exp Mar Bio Ecol. 2009;373(2):87–95. 10.1016/j.marpolbul.2010.04.018 20739251

[65] Ferraro D, Oesterheld M. Effect of defoliation on grass growth. A quantitative review. Oikos. 2002;1:125–33.

[66] Pedersen O, Colmer TD, Borum J, Zavala-perez A, Kendrick GA. Heat stress of two tropical seagrass species during low tides–impact on underwater net photosynthesis, dark respiration and diel in situ internal aeration. New Phytol. 2016;1–12 10.1111/nph.13900 26914396

